# Development of video-based educational materials for kidney-transplant patients

**DOI:** 10.1371/journal.pone.0236750

**Published:** 2020-08-03

**Authors:** Suhee Kim, Man Ki Ju, Sunyoung Son, Soyoung Jun, Sun Young Lee, Chang Sook Han

**Affiliations:** 1 School of Nursing and Research Institute of Nursing Science, Hallym University, Huncheon-si, Gangwon-do, South Korea; 2 Department of Surgery, Gangnam Severance Hospital, Yonsei University College of Medicine, Seoul, South Korea; 3 Department of Surgery, Severance Hospital, Yonsei University College of Medicine, Seoul, South Korea; Imperial College Healthcare NHS Trust, UNITED KINGDOM

## Abstract

**Introduction:**

Treatment adherence has been evaluated as a major predictor of long-term outcome, and education has been suggested to improve adherence. Considering the characteristics of adult learners, it is necessary to implement educational programs that meet the needs of transplant patients. Multimedia education may be well-suited for this. This study aims to develop video education materials in accordance with transplant patients’ self-care needs.

**Methods:**

This study includes a literature review and patient interviews aimed at developing video education materials for the self-care needs of patients who underwent renal transplant surgery at a university hospital in Seoul. Ten patients were interviewed about the desired educational content, accessibility, and other preferences. After verifying the validity of the data, the video scenarios were produced and satisfaction surveys were conducted.

**Results:**

Eleven self-care education items were identified through interviews with 10 kidney transplant patients. The expert validation of video-based educational content result was high (mean CVI = 0.94). The mean score of the patients’ satisfaction evaluation of the completed 7-minute video instructional materials was also high (4.55 on a 5-point Likert scale).

**Conclusion:**

Findings indicate that the video education materials will meet the needs of adult learners and mitigate the limitations of the existing education programs by increasing interest and motivation and may contribute to increased treatment adherence and ultimately, positively effect self-care for new transplant patients.

## Introduction

Globally, an aging population and greater prevalence of chronic disease has increased the number of patients with end-stage renal disease (ERSD) who require transplantation or dialysis [1]. The former procedure is considered the gold standard because it preserves 70–80% of normal kidney function, whereas dialysis only preserves 15–20%. Moreover, transplantation allows patients to freely engage in physical activity, thus elevating patient satisfaction [2]. South Korea performs around 2,000 kidney transplantations annually; 2,108 were performed in 2018 and 2,293 in 2019 [3]. These numbers are about 1.8 times higher than those of 1,142 and 1,238, performed in 2008 and 2009, respectively; kidney transplantations are expected to continue to increase in the future [3]. Furthermore, kidney transplantation rates are projected to continue increasing.

Post-transplantation survival rates are improving with the advancement of surgical techniques and immunosuppressants, leading to a decrease in drug-related adverse effects [4]. Nevertheless, patients who undergo kidney transplantation are associated with serious risks, including rejection, cardiovascular disease, and infection. Thus, they require persistent postoperative management, including strict regulations for medication and diet. Kidney transplants largely fail due to treatment noncompliance, which negatively influences quality of life for transplant recipients [5]. One study on compliance among transplant recipients reported noncompliance by 20–37% of adult patients [6]. Treatment noncompliance is associated with 50% of acute rejection and 15% of organ loss, causing negative short- and long-term physical effects, along with economic losses [6, 7]. Clearly, programs that improve compliance should be a critical part of clinical management for promoting patient well-being. To that end, numerous studies have investigated intervention programs to improve treatment compliance [8, 9]. In South Korea, several studies have examined the effects of educational interventions for kidney-transplant patients [10, 11], but they have focused largely on knowledge transfer. To the best of our knowledge, no one has attempted to develop a protocol that could potentially induce changes in treatment compliance.

Recently, there has been growing interest in educational videos or other multimedia that enhance knowledge retention and stimulate interest, even without the physical presence of an instructor. As an example, a multimedia dietary education program for gastrectomy patients successfully improved their nutritional status [12]. Additionally, audiovisual education on TV or video was more effective at altering behavior than spoken or written education for glaucoma patients [13]. Video-based education reduced repetitive time consumption of nurses caring for hemodialysis patients while simultaneously also maintaining patient compliance [14]. Thus, the pedagogical effectiveness of multimedia is equal or superior to conventional methods, while also being transferable across different locations and situations [15]. In clinical settings, for instance, video-based programs can provide valuable information to patients despite the shortages in personnel and time that preclude traditional educational methods [12]. Given the link between education and treatment success, kidney-transplant patients would greatly benefit from the development of practical multimedia educational materials.

The objective of this study was to develop an educational program that can improve patient self-management compliance after kidney transplantation. Specifically, we first aimed to determine the difficulties experienced by kidney-transplant patients during treatment compliance, along with the probable causes. Next, we aimed to create informational videos that could provide the knowledge required for improved treatment compliance.

## Methods

### Study design

The research used multiple descriptive case studies to investigate the educational demands of patients undergoing outpatient care after kidney transplantation. Data from the case studies were then used to formulate video-based educational materials.

### Procedures

#### Literature review

A literature search was conducted on the educational demands of kidney-transplant patients. Specifically, we analyzed text education materials or YouTube video data provided online by domestic and international hospitals and organizations (Korean transplant society, Seoul National University Hospital, Severance Hospital, Jackson Memorial Hospital health system, Nebraska medicine, UW medicine, ITNS, UHN) to kidney transplant patients.

#### Survey of educational demands among kidney-transplant patients

After receiving approval from the IRB at the hospital affiliated with Y—University in Seoul (Project No.: 3-2017-0312), we conducted 30–40-minute interviews between March 1 and May 31, 2018 with patients receiving post-kidney transplant care at the surgical outpatient department. We extracted a random sample of 10 kidney-transplant patients and obtained their consent before interviews. We amended and supplemented the questions during the interviews used in the research by Kim [16]. Based on this, semi-structured questions were formulated and used in this study. Interviews were conducted one-on-one in the counseling room to protect the privacy of the subjects and were recorded after obtaining consent. The interview was conducted by one researcher. Three questions were asked: knowledge related to disease, contents of drug use, and questions about life affecting treatment (S1 Appendix).

#### Construction and expert validation of educational videos

Based on the survey results and the literature review, educational scenarios were developed and filmed. Content validity was verified by an expert panel consisting of one board-certified transplant surgeon, three nurses with at least 5 years of experience (one coordinator, two nurse specialists), and one professor at a college of nursing. Each educational item was scored for suitability on a four-point scale; items with a content validity index (CVI) ≥ 0.80 were selected.

#### Video production

Production included image or video capture and narration recording. To improve the reliability of the information obtained during interviews, a transplant surgeon participated directly in screening the content. An active production director and two filming experts assisted with filming and editing. The finalized video was produced in Premiere Pro and lasted 7 minutes and 37 seconds (https://www.youtube.com/watch?v=qYu9T-01OpU or https://www.youtube.com/watch?v=-hiOTmgj3UQ).

#### Satisfaction assessment for video materials

The same 10 kidney-transplant patients who participated in the survey were also asked about areas for improvement and overall satisfaction with the resultant educational videos. Because this part of the study focused on using feedback to polish the educational materials, there was no need to calculate the number of subjects needed for statistical analysis. Satisfaction assessments were conducted between August 1 and September 31, 2018. To protect patient privacy, the video viewing and questionnaire completion occurred in an outpatient treatment room, one person at a time. In addition to the time required for watching the video, interview-based feedback and satisfaction assessment took 10 minutes.

The satisfaction questionnaire was an amended version of an instrument for assessing video programs [17]. Items (content difficulty, language suitability, interest, motivation, effectiveness, duration appropriateness, screen-composition suitability, audio quality, and picture quality) were scored on a five-point Likert scale, with higher scores indicating greater satisfaction. We also received verbal feedback on the video content by the patients.

## Results

### Literature review

Currently, in Korea, medical personnel provide education on self-care after kidney transplantation before discharge, and thereafter provide regular group education or online textual education materials presented by hospitals and transplantation societies. The number of patient education data produced by each hospital and posted on YouTube has recently started to increase, but the number has not yet been large.

Most educational materials include information about drugs (immunosuppressants, antiviral drugs, other drugs), complication management (rejection reaction, infection, diabetes after transplantation), nutrition-management, daily life-management (prevention of infection, work life, daily life, exercise, travel, Immunization, regular checkups, etc.) were assessed (S2 Appendix).

### Subject characteristics

Participants (six men, four women) were 25–62 years old (mean = 42.6 years). Pre-transplant diagnoses for the participants included hypertension (four patients), immunoglobulin A (two patients), diabetes mellitus, systemic lupus erythematosus, rapidly progressive glomerulonephritis, and polycystic kidney (1 patient each). Time since transplant ranged from 0.6 to 8.3 years (mean = 5.2 years; Table 1).

**Table 1:** Participant characteristics (N = 10).

Patient	Gender	Age	Pre-Diagnosis	Post-Kidney transplantation period (in years)
**1**	M	55	IgA	**1.8**
**2**	M	62	HTN	**3.5**
**3**	F	30	HTN	**5.3**
**4**	F	46	DM	**5.1**
**5**	M	33	HTN	**7.9**
**6**	F	25	RPGN	**7.2**
**7**	F	39	SLE	**0.6**
**8**	M	40	IgA	**8.3**
**9**	M	39	HTN	**4.3**
**10**	M	57	PCK	**8.3**
**Mean**		**42.6**		**5.23**

HTN (hypertension), IgA (immunoglobulin A), DM (diabetes mellitus), SLE (systemic lupus erythematosus), RPGN (rapidly progressive glomerulonephritis), PCK (polycystic kidney).

### Educational demands survey

#### Educational objectives and methods

Based on patient interviews, we clarified the desired educational objectives, as well as duration, medium, and frequency of educational materials. Patients desired information about post-transplant kidney management that would allow them to use the transplanted kidney for as long as possible. They preferred receiving educational content through a cell phone, because this option places fewer restrictions on both location and frequency of access (Table 2).

**Table 2:** Patient interview results (educational objectives and methods).

Content	Interview statement
Educational objectives	*“Our primary interest as transplant recipients is how long we can use the kidney*. *We want information about the actions and precautions required to increase kidney longevity*.*”*
Education duration and medium	*“The video can be longer than 3 min*. *If you are interested enough*, *you will watch it however long it is*. *Watching it on a cell phone is the best*, *because then you can watch it anywhere*.*”*
Education frequency	*“I think it is important to receive education frequently*, *not just once*. *When I left the hospital*, *I was not able to focus on anything and I do not remember much*, *so giving me information at that time is not helpful*. *Also*, *you start to become lax about following treatment after more time passes*.*”*

#### Educational content

Patient interviews revealed that they wanted specific explanations regarding issues that could arise due to treatment noncompliance, which they felt would motivate post-transplant self-management. Specific items were preferred as educational content included post-kidney transplant rejection and coping methods, importance of taking immunosuppressants, as well as post-transplant dietary management (Table 3). In addition, educational demand was present for “other emergency medication,” “managing complications,” “coping skills for emergency situations,” and “social welfare information.”

**Table 3:** Results of patient interviews to determine educational content.

Content	Interview statement
Results of treatment noncompliance	*“I think it would be good to talk about the harm of not complying with treatment*. *For example*, *how much of an effect is there if we comply or do not comply*, *like pointing out that there is a higher risk of diabetes in patients who do not comply*. *You should explain how quickly the kidney becomes damaged*, *so that we understand why we have to exercise and watch what we eat*.*”*
Rejection and coping methods	*“It is difficult and scary*, *you know*, *because rejection could happen at any time; there is no time limit*. *Is this a symptom of rejection…*? *If I feel under the weather*, *is that fatigue or rejection… Right now*, *if I get a fever*, *I go to the emergency room at once*. *I want to be well-informed so I know how to cope with rejection*.*”*
Importance of taking immunosuppressants	*“I think that once I am used to life after the transplant*, *I will fall into bad habits*. *I will forget to be grateful*, *and medication becomes a burden*. *How am I supposed to take this medicine every day*? *That is the kind of feeling I am talking about*. *That is why I think it is crucial to explain the importance of medication*.*”*
Dietary management post-transplantation	*“When I am discharged after surgery*, *lifestyle-related things like food… there is a little bit of information in the leaflet*, *but once I start eating*, *I start to have questions*. *If I try to look at books on dietary management*, *one book says do not eat this and another book says eat a lot of it; I do not know what advice to follow*.*”*
Other emergency medication	*“So*, *I want you to tell me some medicines I can take*. *I want you to tell me what medications to take when I have a body or cold*. *For example*, *if I have a headache*, *if I have a cold*, *or if I have allergies*, *I would like the education to clearly tell me which medicine to take*.*”*
Managing complications	*“In fact*, *I did not know that urinary tract infections can develop so well*. *At first*, *I did not know if it was a urinary tract infection*, *I called an ambulance*, *and I came to the emergency room*.*”*
Coping skills for emergency situations	*“I was not sure how to deal with it when I got hurt*. *First of all*, *I do not know if I can use the medicine*, *and what kind of medicine I will use to prevent my kidneys from being overwhelmed*.*”*
Social welfare information	*“All the information is helpful to see here now*. *But later on*, *it seems to be a little more helpful to register a disabled person*, *buy a car*, *and pay taxes*.*”*

### Construction and expert validation of video-based educational content

Mean CVI was 0.94. The lowest CVI (0.85) was for post-transplant mental health and quality of life, social welfare information, and other emergency medications. The highest CVI (1.00) was for post-transplant daily living, coping skills for emergency situations, immunosuppressant medication, meals, managing complications, as well as rejection and treatment. We selected all items with CVI ≥ 0.80 (Table 4).

**Table 4:** Construction and expert validation of video-based educational content (N = 5).

Content	CVI
Post-transplant mental health and quality of life	0.85
Post-transplant daily living: occupation, exercise, sexual activity, pregnancy	1.00
Postoperative management (outpatient visitation procedure)	0.90
Regular health examinations	0.90
Social welfare information	0.85
Coping skills for emergency situations	1.00
Immunosuppressant medication	1.00
Meals after kidney transplantation	1.00
Managing complications	1.00
Other emergency medication	0.85
Rejection and treatment	1.00
**Mean**	**0.942**

### Satisfaction assessment for video-based educational materials

The results of satisfaction assessment revealed that patients strongly agreed with “The picture quality was good” (5 points), whereas “Watching the educational content as a video was entertaining” earned the lowest score (4 points; Table 5). The mean score was 4.55 points, and all nine categories scored ≥4 points, demonstrating high satisfaction in the video-based educational materials. In addition, participants reported liking the strong presentation of the importance of health care at the beginning of the video. They also liked the explanation of side effects and the precautions about daily life.

**Table 5:** Satisfaction assessment for video-based educational materials (N = 10).

Category	Item	Mean score
Content difficulty	The video content was easy to understand.	4.5
Suitability of language	I thought the language used was appropriate.	4.75
Interest	Watching the educational content as a video was entertaining.	4
Motivation	I became more interested in post-transplant management.	4.5
Effectiveness	I will diligently adhere to the post-transplant management at home, as shown in the video.	4.75
Suitability of duration	The video duration was appropriate.	4.25
Suitability of screen composition	I thought the images used were appropriate.	4.5
Picture quality	The picture quality was good.	5
Audio quality	The audio quality was good.	4.75
Overall mean		**4.55**

## Discussion

In this study, we succeeded in developing a video-based education program through an educational demands survey administered to kidney-transplant patients. Our interviews revealed that patients were most interested in transplant rejection and methods of coping. Patients expressed alarm at the idea of experiencing rejection-related complications at home and therefore, desired the relevant educational content to be delivered beforehand in a manner that facilitated free access at any time. Additionally, patients reported that they had trouble remembering the provided information on discharge because they were disoriented during that period. Patients also reported that their personal interest in the content meant they would tolerate longer videos (i.e., over 3 mins), so we took this feedback into consideration when deciding on video length. The developed video scenarios were based on patient reports of interest in how long they can use the kidney and the effects of following or not following the educational content. The latter point, in particular, is in line with adult learning theory, suggesting that “when adults learn something, they invest considerable effort in identifying the benefits they obtain from learning and the potential negative consequences of not learning” [18]. Our video is now being used for education on patient discharge and has had over 5,000 views on YouTube in the 16 months since being uploaded (November 8, 2018).

Our study had several limitations. First, we did not include any measurement tools that would indicate whether compliance was improved, so we did not verify whether treatment compliance actually increased. In addition, it was confirmed only immediately after education how well the patients understood the education contents. To determine treatment compliance, sustained actions post-discharge are more important than actions performed immediately after the discharge. Thus, in a future study, we plan to evaluate how our video influenced patient behavior, and how long the understanding of education lasted. Second, though we conducted patient interviews to identify the educational demands, the responses have not been evaluated using a properly structured qualitative analysis. Third, our video is currently only available in Korean; videos in other languages are necessary to accommodate the increasing number of international transplant patients. Finally, our video may not be useful for older people who are not familiar with the latest technology. Therefore, there is a need for follow-up studies to increase the treatment performance of the elderly after kidney transplantation.

In spite of these limitations, we believe we succeeded in delivering quality educational content that patients actually needed through the combined use of literature review and interviews. We also increased accessibility by making the video available online, allowing patients access anywhere at any time. Our addition of subtitles ensured accurate delivery of content and improved understanding from viewers. Finally, we used simple terminology that can be readily understood by the general public, so that the patient’s family or colleagues can view the materials and help the patient to maintain the health of the transplanted kidney.

## Conclusions

We developed video-based educational materials customized to meet the educational demands of kidney-transplant patients. We overcame the limitations of previous educational programs implemented at organ transplant centers, including restrictions on access, burden on medical personnel, and low patient interest. We believe this educational material will increase treatment compliance and therefore, improve quality of life among transplant patients. Given these promising results, we recommend the development of more video-based educational materials that account for variation in health literacy among patients.

## Supporting information

S1 Appendix (DOCX)

S2 Appendix (DOCX)

S3 Appendix (DOCX)
