# Supplementary material for: Development of video-based educational materials for kidney-transplant patients
Source: PLoS One. 2020 Aug 3;15(8):e0236750. doi: 10.1371/journal.pone.0236750 (PMC7398512; doi:10.1371/journal.pone.0236750)
Supplement: S1 Appendix — (DOCX) [file pone.0236750.s001.docx]

S1 Appendix. The semi-structured questionnaires

1. Disease-related knowledge

- As you prepared for the transplant? Have you received enough information from your medical staff to ask about your questions?
- Have you fully understood, if you have been told by the medical staff, about the transplant?
- What information do you currently want to receive from your healthcare provider about the transplant procedure?

1. Questions about medication use

- What are your questions or inconveniences while using immunosuppressants?
- Have you heard about immunosuppressants?
- What did you want your medical staff to explain about taking immunosuppressants?
- What information do you currently want to receive from your healthcare provider about immunosuppressants?

1. Questions about life-affecting treatment

- What are your questions about precautions in everyday life?
- What aspects about reduced immunity do you care about in your daily life?
- What information did you receive from the medical staff about the precautions in everyday life and did you fully understand it?
- What information do you currently want to receive from medical staff?
