# Supplementary material for: Development of video-based educational materials for kidney-transplant patients
Source: PLoS One. 2020 Aug 3;15(8):e0236750. doi: 10.1371/journal.pone.0236750 (PMC7398512; doi:10.1371/journal.pone.0236750)
Supplement: S2 Appendix — (DOCX) [file pone.0236750.s002.docx]

**S2. Appendix. Literature review analysis table**

| **Main Category** | **Sub-category** | **Subclass** | **A** | **B** | **C** | **D** | **E** | **F** | **G** | **H** |
| --- | --- | --- | --- | --- | --- | --- | --- | --- | --- | --- |
| Drugs | Immunosuppressants | Effects and side effects of immunosuppressant types | O | O | O | O | O | O | O | O |
|  |  | Precautions when taking immunosuppressants (time to eat, effects, side effects) |  | O | O | O | O | O | O |  |
|  | Antiviral drugs | Antiviral drugs | O | O |  |  |  | O |  |  |
|  | Other drugs | Blood pressure medicine, diabetes medicine, aspirin, antacid, multivitamins | O | O |  | O |  | O |  | O |
|  | Emergency medication | Over the counter medication | O | O |  |  |  |  | O | O |
| Complications | Rejection reaction | Signs and symptoms | O | O |  |  | O | O | O |  |
|  |  | Treatments | O | O |  |  | O | O | O |  |
|  | Infection | Post-transplant infection | O | O | O |  | O | O | O |  |
|  |  | Types of infection, symptoms and treatment | O | O | O |  |  | O |  |  |
|  | Diabetes after transplantation | Diabetes | O |  |  | O |  | O |  |  |
| Test results | Blood test results | WBCs, BUN, Creatinine | O |  |  | O | O | O | O |  |
|  |  | Immunosuppressive blood concentration | O |  | O | O | O | O | O |  |
| Nutrition | Nutrition management | Precautions when dieting by nutritional ingredients | O | O |  | O | O | O | O | O |
|  |  | Food safety | O | O |  | O | O | O | O | O |
|  |  | Heart healthy eating | O | O |  | O | O | O | O | O |
| Hospital checkups | Outpatient regular checkup | Outpatient examination and process | O | O | O | O | O | O | O |  |
|  | Emergency | Emergency situations where you need to contact the medical staff | O | O | O |  | O | O | O | O |
|  |  | Emergency contact | O | O | O |  | O |  | O | O |
| Daily life |  | Prevention of infection | O | O | O | O | O | O | O | O |
|  |  | Skin and wound care | O |  | O |  |  |  |  | O |
|  |  | No smoking and drinking | O | O | O |  | O | O | O |  |
|  |  | Exercise | O | O | O | O | O | O | O |  |
|  |  | Sex life | O |  | O | O | O | O | O | O |
|  |  | Emotional change | O |  |  | O |  |  |  |  |
|  |  | Vacation and travel | O | O |  | O | O | O | O | O |
|  |  | Pets | O |  | O | O |  | O |  |  |
|  |  | Vaccination | O |  | O | O | O | O | O | O |
|  |  | Flower management | O |  | O |  |  |  |  |  |
|  |  | Work life |  | O |  | O | O | O | O | O |
|  |  | Daily life |  | O | O | O | O | O | O | O |
|  |  | Pregnancy & Contraception |  | O | O | O | O | O | O | O |
|  |  | Regular checkups: health checkups, cancer checkups, dental checkups | O | O |  | O | O | O | O | O |
|  |  | Where to get information | O | O | O | O | O | O | O |  |

A. Jackson memorial hospital health system: Teaching Transplant Journal

B. Nebraska medicine Kidney Transplant patient education material

C. UW medicine: University of Washington medical center. Kidney transplant patient education material

D. ITNS: International Transplant Nurses Society

E. Severance Hospital

F. Korean transplant society

G. Seoul National University Hospital

H. UHN patient education (YouTube): Lifestyle adaptations for transplant patients
