# Supplementary material for: Development of video-based educational materials for kidney-transplant patients
Source: PLoS One. 2020 Aug 3;15(8):e0236750. doi: 10.1371/journal.pone.0236750 (PMC7398512; doi:10.1371/journal.pone.0236750)
Supplement: S3 Appendix — (DOCX) [file pone.0236750.s003.docx]

**S3 Appendix. Overview of the video-based educational materials**

Title: Health care after transplantation of kidney transplant patients

| Time | Content |
| --- | --- |
| 00:00 ~ 01:00 | Importance of immunosuppressants and health care after transplantation   - Malfunction of the transplanted kidney - Irregular medication, non-compliance with health care guidelines -> Increased rejection rate, increased complications, decreased lifespan of transplanted kidney |
| 01:01 ~ 02:07 | Care guidelines for maintaining post-transplant health  1. Know and take immunosuppressants well.   - Description of rejection - The role of immunosuppressants - Taking medication at the prescribed dose at the right time - What if you accidentally forget to take medicine - Food to avoid: Grapefruit juice, red ginseng, etc. |
| 02:08 ~ 02:59 | 2. Be careful not to get infected.   - Increased risk of infection from immunosuppressants - Notes for preventing infection: Hand washing & regular gargling, wear a mask when going out, avoid eating raw seafood or raw meat, etc. |
| 03:00 ~ 04:05 | 3. Must be aware of and respond to rejection.   - Description of rejection - Symptoms of rejection suspected: Fever of 38.3 degrees or more, edema of the transplant site, tenderness, hematuria, changes in urine volume, weight gain, edema, chills, pain, headache, dizziness, nausea, vomiting, etc. - What to do if symptoms are found |
| 04:06 ~ 05:07 | 4. Healthy diet management.   - For a quick recovery of kidney function - To prevent weight gain, hyperlipidemia, hypertension, diabetes, and osteoporosis - Sufficient water intake of 1.5 to 2 liters per day - Good protein intake - Consume less salt - Increased calcium intake, etc. |
| 05:08 ~ 05:27 | 5. Regular outpatient checkups are required.   - Regular outpatient check-ups are a must - At the time of examination, the test will confirm whether a rejection reaction has occurred and the appropriate dose of the immunosuppressant. |
| 05:28 ~ 07:21 | 6. Understand other precautions in everyday life.   - Work and school can be returned to 3 months after surgery - Sexual relations are possible when the body recovers after surgery. It is important to prevent infection and contraception during sex. If you have a child plan, you should talk to your doctor because you need to control the immunosuppressant. - When going out, use sunscreen, long sleeves, and a hat to prevent skin cancer - Work out slowly after transplantation, but please continue. - It is not advisable to keep pets, and if they are raised, immunization should be done thoroughly, etc. |
| 07:22 ~ 07:38 | Finish greeting |
